# Cannabis, Cannabidiol Oils and Tetrahydrocannabinol—What Do Veterinarians Need to Know?

**DOI:** 10.3390/ani11030892

**Published:** 2021-03-20

**Authors:** Nancy De Briyne, Danny Holmes, Ian Sandler, Enid Stiles, Dharati Szymanski, Sarah Moody, Stephan Neumann, Arturo Anadón

**Affiliations:** 1 Federation of Veterinarians of Europe (FVE), 1040 Brussels, Belgium; sar_email_ahs@yahoo.co.uk; 2 Holmes St Anthony’s Veterinary Hospital, St Anthonys, Caherslee, V92 V6YK Tralee, Ireland; holmesveterinary@gmail.com; 3 Canadian Veterinary Medical Association (CVMA), Ottawa, ON K1R 7K1, Canada; isandler@greywolfah.com (I.S.); enid.stiles@gmail.com (E.S.); 4 American Veterinary Medical Association (AVMA), Schaumburg, IL 60173, USA; DSzymanski@avma.org; 5 Companion Animal Clinic, Institute of Veterinary Medicine, University of Goettingen, 37073 Göttingen, Germany; sneuman@gwdg.de; 6 Department of Pharmacology and Toxicology, Faculty of Veterinary Medicine, Universidad Complutense de Madrid, 28040 Madrid, Spain; anadon@vet.ucm.es

**Keywords:** cannabis, cannabinoids, cannabidiol (CBD), CBD oils, Marinol, THC (Delta-9-trans tetrahydrocannabinol or tetrahydrocannabinol), veterinary use, veterinary medicine, companion animals, toxicity, dog, cat, horse

## Abstract

**Simple Summary:**

As cannabis-derived products have become more available for human medical and recreational use, veterinarians are seeing more cases of cannabis toxicosis in pets. In addition, animal owners have increasing interest in giving these products to their pets. These clients understandably are asking, “Are these products legal, safe, and effective for treating medical conditions in animals?”. This review considers the different types of cannabis and cannabis-derived products, historical examples of use in animals, the industry, the existing framework of legislation and regulations for use in humans and animals as medicines and/or supplements, veterinary clinical use of these products, toxicosis, and recommendations and warnings around their veterinary use.

**Abstract:**

As cannabis-derived products have become more available, veterinarians are seeing more cases of toxicosis. In addition, animal owners are having an increasing interest in using these products for their pets. This review looks at the situation in Europe and North America, the different types of cannabis and cannabis-derived products with historical examples of use in animals, and the cannabis industry. The existing regulatory framework for use in humans and animals as medicines and/or supplements was examined. Finally, a review of the clinical indications for which medicinal cannabis is authorised, a discussion of toxicosis, and recommendations and warnings around medical cannabis use are presented.

## 1. Introduction

The use of Cannabis sp. and Cannabis-derived products is increasing globally. Concurrently, scientific interest has grown with cannabidiol (CBD)—related citations in PubMed increasing from 40 in 2000–2002 to 458 in 2014–2016 [1]. In North America and Europe, many countries have passed legislation permitting the medicinal use of certain cannabis-derived products in humans, and some countries have passed laws permitting recreational use.

As cannabis-derived products have become more available, veterinarians have seen more cases of toxicosis [2] and increased interest among clients in therapeutic use of these products for their companion animals [3]. Clients ask questions about the legality, safety, and effectiveness of cannabis-derived products for treating medical conditions in animals.

Although cannabinoids such as CBD may have potential for therapeutic promise, scientific evidence supporting their use in animals is currently limited and few well-controlled studies exist, most of which focus on companion animal use.

## 2. Definitions

Cannabis is an Asian herb of the family *Cannabaceae*—the hemp family, that has tough fibre and is often separated into a tall loosely branched species (*Cannabis sativa*) and a low-growing densely branched species (*C. indica*) [4,5]. Unfortunately, the term is used interchangeably in popular culture with marijuana although strains of cannabis can be either marijuana or hemp depending on their concentration of THC (delta-9-trans tetrahydrocannabinol).

Hemp (*Cannabis sativa*) is legally defined in the United States (US) [6] and European Union (EU) as any part of the cannabis plant that contains less than or equal to 0.3% THC on a dry weight basis. Hemp has traditionally been farmed for industrial uses (e.g., textiles, paper, biodiesel, constructions materials), as well as for food (hemp seeds and hemp seed oil). Typically, hemp contains relatively high amounts of non-psychoactive cannabinoids. In the US, hemp is not legally recognized as a dietary supplement for either people or animals, nor as a feed supplement for animals, so products labelled as such are illegally marketed [7]. Certain varieties of the hemp plant are legally grown in EU under license. The varieties of hemp permitted to be grown are those listed in the EU’s “Common Catalogue of Varieties of Agricultural Plant Species” [8]. The term used to describe these varieties is “Industrial Hemp”.

Marijuana refers to a mixture of cut, dried, and ground flowers, leaves and stems of leafy green cannabis plant. The term “Marijuana” is typically used for the psychoactive dried resinous flower buds and leaves of the cannabis plant (*C. sativa* or *C. indica*), but can refer to any part of the cannabis plant that contains greater than 0.3% of THC [9]. Marijuana may be smoked, vaped, or ingested (e.g., baked goods) in some countries especially for its intoxicating effect [10]. In some countries, home-made hash oil (also called honey oil or butane hash oil) is also popular. This potent oil is obtained by the extraction of cannabis using an organic solvent, often butane or n-butane [11]. Marijuana is typically classified as a Schedule 1 drug in EU countries and in the US, based on the UN Treaties on Psychoactive Substances of 1961 (revised 1971) [12]. In informal discussions, “marijuana” and “cannabis” are both terms used to describe the dried leaves and flowering heads of the cannabis plant. Subdivisions of the terms are Medical Marijuana or Medical Cannabis and Recreational Marijuana or Recreational Cannabis, despite the fact that cannabis may be marijuana or hemp depending on its THC concentration (measured on a dry weight basis). In some countries, like Canada, the term Cannabis is now used to reference all types of Cannabis to remove the stigma of the term Marijuana [13].

Cannabinoids are any of the various naturally occurring, biologically active, chemical constituents of the cannabis plants that bind to cannabinoid receptors (See Section 9 “Pharmacokinetics”). Over 480 cannabinoids and other substances have been isolated [14]. The amount of each substance contained in a sample of cannabis depends on the subspecies, the age of the plant, the time of year the leaves were harvested, the way they have been dried, and other factors [15]. During the growth of the plant, they are present in the acid forms THCa and CBDa, which through “decarboxylation” by heating transform in THC and CBD [16]. While most research has been done on the medical qualities of CBD and THC, further research is starting to look at other components of the hemp plants, such as THCa, CBDa and cannabigerol (CBG) oil [17,18].

Delta-9-trans-tetrahydrocannabinol (Δ9-THC, more commonly called tetrahydrocannabinol (THC), is the principal psychoactive constituent of marijuana [19]. THC is a lipid, assumed to be involved in the plant’s self-defence against insect predation [20], ultraviolet light [21,22], and environmental stress [23]. The concentration of THC found in plants depends on environmental conditions, including amounts of light, moisture, soil type, pH, nutrients, and trace minerals [24].

THC is the psychoactive chemical that gives marijuana performance as a recreational drug. Different components of the cannabis plant yield between 5 and 20% THC, the highest concentration produced largely by plant hairs (trichomes) and the female flower [25]. Traditionally, marijuana varietals of *Cannabis sativa* plants have up to 10% THC [26].

Cannabidiol (CBD) is generally made from the *Cannabis sativa* L. plant [17,27]. The plant contains hundreds of different active compounds and of these, more than 100 are cannabinoids which, depending on the compound, have either psychoactive or non-psychoactive effects. CBD is a non-psychoactive lipid cannabinoid [19]. CBD has been used in human medicine to mitigate anxiety [28], improve appetite [29], relieve nausea [30], control seizures of certain types [31], and assist in the management of sleep disorders [32]. Assorted CBD products are available throughout Europe for human use both online and through dispensaries [33]. A similar scenario exists in the US where CBD products are also widely available and often marketed for therapeutic purposes. To legally market such products, claims of safety and efficacy must be substantiated through the federal government approval process. If such CBD products are not granted such approval, they cannot be legally marketed and, with one exception, all products in the US that have such a therapeutic claim have not been approved [7] (See also Section 5 “The Industry of Cannabis in North America” and Section 6 “Regulatory Framework in the EU and North America”).

Resin comes from the dried leaves (‘marijuana’), the more potent female flowering heads of the plant (‘sinsemilla’), or the sticky cannabis resin that can be smoked or consumed in a variety of foodstuffs. Several cannabinoids are present in the plant resin, but THC is considered the most active and main psychoactive agent. Hashish is the resin extracted from the top of the flowering plant and is higher in THC concentration than marijuana.

## 3. The History of Cannabis Use in Veterinary Medicine

### 3.1. Horses

Greek writers reported the use of cannabis for dressing sores and wounds in humans and horses [34]. The dried leaves were used against nosebleeds and the seeds against tapeworms. The green seeds were steeped in a liquid such as water or a variety of wine, pressed out, warmed and then instilled into the ear for pains and inflammation associated with blockages [34]. The “Berlin Hippiatrica”, a collection of horse remedies, indicates the chopped leaves were used to dress a wound. First some vinegar and pitch were brought to a full rolling boil, then wax, mustard, wheat-chaff, and roasted pine-resin were added, and the resulting mixture (presumably cooled) was applied liberally, then chopped cannabis leaves and grass trimmings were put on top before the wound was bound [35].

Another collection, the “Cambridge Hippiatrica” offers a recipe for the treatment of tapeworms for horses [35]. Until relatively recently, cannabis was found in a large number of veterinary medications designed to treat colic, spasmodic colon, and other ailments in equine patients [36,37]. The bottles of some of these veterinary drugs survive to provide us with evidence of the therapeutic uses of this ubiquitous plant. The earliest recorded known example was from 1607 when the author Edward Topsell claimed that mixing hemp seeds with a horse’s regular ration encouraged rapid weight gain [38]. In the 1800′s American veterinarians routinely prescribed equine colic medication which contained high doses of marijuana. O’Reilly notes that the Parke Davis pharmaceutical company was one of the US’ leading suppliers of top quality “liquid cannabis” [36]. The company, having collected hemp seeds from India and Nepal at the start of the 20th century, began growing high-grade marijuana in Michigan and the Blue Ridge mountains [39]. The US Department of War had no hesitation in recommending its use for colic. Its 1915, “Manual for Farriers, Horseshoers, Saddlers and Waggoners” recommended giving colicky horses “One teaspoon of liquid Cannabis Americana mixed with one tablespoon of olive oil” [40].

### 3.2. Dogs

Whilst in India in 1843, Irish Physician William O’Shaughnessy noted the widespread use of Indian hemp for a “multitude of afflictions”, he was unable to “trace any notice of the employment of this drug in Europe” [41,42]. From his research, he concluded that “there was sufficient evidence to show that hemp possesses, in small doses, an extraordinary power of stimulating the digestive organs, exciting the cerebral system, of acting also on the generative apparatus [41]. The influence of the drug in allaying pain was equally manifest in all the memoirs referred to.” Inspired, O’Shaughnessy initiated his own studies in animals, administering “majoon”, an Arabic cannabis sweet, to a dog and reporting that “he ate it with great delight” and became “ridiculously drunk” [41]. Similarly, another dog was given “churrus”, a Nepalese hemp resin, and O’Shaughnessy reported he became “stupid and sleepy, dozing at intervals, starting up, wagging his tail, as if extremely contented; he ate some feed greedily; on being called to, he staggered to and fro, and his face assumed a look of utter helpless drunkenness”. In all cases, the animals recovered and were “well and lively” after a few hours. [41].

### 3.3. Cats

A systematic review of the literature retrieved no information on any historic clinical use of cannabis in cats.

## 4. The Cannabis Industry in the EU and North America

### 4.1. Europe

Over EUR 500 million were invested in the European cannabis industry in 2018. Europe’s cannabis market is estimated to be worth up to EUR 2.4 billion by 2024, mostly made up by medical cannabis use. Germany is currently the largest European market for medical cannabis use, and the third largest market worldwide after the US and Canada. Having seen increased demand in medical cannabis use, many European countries are now trying to boost domestic production [43,44]. No data are available on the size of the cannabis industry in Europe related to animals, but it is estimated to be very small.

Although the legal cannabis market in Europe is targeted strictly towards medical consumers, the consumption of hemp-derived CBD-infused products for non-medicinal purposes is legally permitted across much of the world. The current market size for CBD in Europe is about EUR 450 million, representing 31% of the global CBD oil market share, [45].

### 4.2. North America: US

In the US, the cannabis industry was estimated at USD 13.6 billion in 2019 [46]. The US recently passed the Agricultural Improvement Act of 2018 [6] that de-scheduled “hemp” (defined as *Cannabis sativa* L with ≤0.3% THC on a dry weight basis). As a result, hemp is no longer subject to the restrictions of the US Controlled Substances Act. Many manufacturers have interpreted this to mean that hemp products can now be marketed for human or animal use with no further federal regulation, which is not the case. The US Department of Agriculture has proposed standards for hemp growers and states that wish to apply for licensure so that the hemp supplied to manufactures for CBD production is of known chemical composition [47]. Manufacturers also must comply with legal requirements for bringing a food or drug to market as described in the US Federal Food, Drug and Cosmetic Act [48]. As is the case for the European industry, many cannabis products in the US are marketed with claims of therapeutic efficacy without having gone through the approval process to substantiate those claims and are therefore being marketed illegally. Adding to the confusion is that additional regulation comes into play at the US state level, which may, or may not, be consistent with federal regulation.

In the current regulatory landscape where there is often much confusion over varying legal frameworks and inadequate resources for widescale enforcement, economic incentives can take precedence over concern for legal consequence [49]. Manufacturers readily market their products to consumers leading to the widescale availability of a variety of cannabis products intended for human or animal use despite their often questionable legal status.

### 4.3. North America: Canada

The Canadian cannabis market has been steadily growing. In 2017, about 4.9 million Canadians aged 15 to 64 spent an estimated CAD 5.7 billion on cannabis for medical and non-medical purposes, which amounts to around USD 1200 per cannabis consumer. The federal government legalized the recreational use of cannabis in 2018. The total cannabis market in Canada, including medical and recreational use of cannabis products, is expected to generate up to CAD 7 billion in sales in 2020 with about half of this coming from the legal recreational drug market [50]. No data are available on the industry of cannabis in the Canada related to animals.

## 5. Regulatory Framework in the EU and North America

### 5.1. EU

#### 5.1.1. Human and Veterinary Medicinal Products

Human and veterinary medicinal products can be authorised in the EU either via a centralised EU system (by the European Medicines Agency—EMA) or by EU national medicines agencies. There is currently no harmonised framework in the EU for the medical or recreational use of cannabis. Some form of medical cannabis is now legal in more than 22 EU countries. Several human cannabis-derived medicinal products have been authorised either via EMA or via EU national medicines agencies (see Table 1). As of February 2020, there are 17 active clinical trials that involve investigating the efficacy of cannabis and cannabinoid medications against diseases ranging from schizophrenia to endometriosis [44].

In 2021, Luxembourg could become the first EU country to legalize recreational use of cannabis for adult use. A further 12 EU countries have decriminalised the recreational drug use of personal amounts of cannabis [51]. In 2019, the European Parliament passed a resolution calling the EU to implement an EU-wide policy for medical cannabis and properly funded scientific research [52].

The criminal or administrative response to drug use offences is the responsibility of the EU Member States, not of the EU. According to Article 168 of the Treaty on the Functioning of the EU, “The Union shall complement the Member States’ action in reducing drugs-related health damage, including information and prevention” [53]. There are some EU laws affecting cannabis trafficking offences. The Council Resolution on cannabis of July 2004 [54] encouraged the EU Member States to take measures against the cultivation and trafficking of cannabis within the Union and to consider taking measures against internet sites providing information on cultivation.

##### Authorisation as Food Ingredient or Additive

This includes any substance intended for consumption, e.g., oils, sweets, animal treats, or food supplements. As per the CBD regulations of the EU, CBD infused products are not illegal in the EU unless they contain more than 0.2% THC. Some EU countries, e.g., Belgium are stricter and have zero tolerance for THC.

##### THC

Article 2(g) of Regulation (EU) No 178/2002 states that human food shall not include: “narcotic or psychotropic substances within the meaning of the United Nations Single Convention on Narcotic Drugs, 1961, and the United Nations Convention on Psychotropic Substances, 1971” [55]. THC is listed as one of these narcotic or psychoactive substances. The safety of THC in foodstuffs has been addressed in 2015 by the European Food Safety Authority (EFSA) which concluded that consuming food containing THC at greater than 1 µg/kg body weight in one sitting, or in a day, may have adverse effects [56].

Generally, hemp/CBD oil (THC < 0.2%), obtained by “cold pressing” the seeds or other parts of the hemp plant, does not require authorisation. This is because hemp oil was consumed in the EU to a significant degree before 1997. If, however, the CBD/hemp oil is subjected to certain forms of extraction or purification techniques, then since January 2019, revised rules mean a “Novel Food Authorisation” may be required, as there may be an accompanying increase in undesirable constituents [57,58]. On November 2020, the Court of EU issued Judgment C-663/18, which says that an EU Member State may not prohibit the placing on the market of CBD which is legally produced in another Member State if it is extracted from the whole plant *C. sativa*, and not only from its fibres and seeds [59].

#### 5.1.2. Animal Feed

Following a request from the European Commission, the Panel on Additives and Products or Substances used in Animal Feed (FEEDAP of EFSA) delivered a scientific opinion on the safety of hemp (*Cannabis* genus) for use as animal feed [60]. Different types of feed materials may be derived from the hemp plant: hemp seed meal/cake, hemp seed oil and whole hemp plant (including hemp hurds, fresh or dried). Further products are hemp flour (ground dried hemp leaves) and hemp protein isolate from seeds. Hemp seed and hemp seed cake could be used as feed materials for all animal species and EFSA defined maximum incorporation rates in the complete feed per species e.g., 3–7% in poultry, 2–5% in pigs for hemp seed and hemp seed cake, 5% in ruminants for hemp seed cake and 5% in fish for hemp seed. Feeding efficacy trials also demonstrate that hemp and its derivates may be included in diets of livestock as a good source of crude protein and essential fats [61,62].

#### 5.1.3. Pet and Equine Supplements

There are many companies in the EU marketplace today selling “nutritional supplement” cannabis-derived products for dogs, cats, and horses, some of which make what clearly appear to be therapeutic feed claims. These products are being promoted as aids for itching, anxiety, nausea, poor appetite, seizures, cancer, digestive problems, inflammation, immune disease, and reduced mobility due to joint pain in animals. It is against the law to make therapeutic feed claims about nutritional products. Under the EU Regulations, products for which therapeutic claims are made must firstly be approved by a National Health Product Agency or EMA, to become a medicine and be legally manufactured and marketed. This provides scientific data about the efficacy and safety of products. Veterinarians cannot offer scientific advice on the effectiveness of a nutritional product to treat a disease, as it is not a medicine and such claims are (a) illegal; (b) unproven; and (c) potentially unsafe. No health claims relating to hemp or CBD are authorized for use under regulation (EC) No 1924/2006 in the EU [63].

### 5.2. North America: US

#### 5.2.1. Human and Veterinary Medicinal Products

When a pharmaceutical sponsor begins drug development and investigates an active substance for therapeutic potential there are many technical requirements necessary to obtain United States Food and Drug Administration (USFDA) approval pertaining to chemistry, manufacturing and controls, target animal safety, human food safety (if applicable), environmental impact, effectiveness and labelling [64]. In the US, certain cannabis-derived substances or their synthetic analogues have been approved for use in humans. These include the most recent approval of cannabis-derived CBD, Epidiolex [65] for the treatment of refractory epilepsy in children. Two synthetic formulations of THC have also been approved: Nabilone, brand name Cesamet [66], which was approved for use as an antiemetic in patients undergoing chemotherapy; and Marinol (Dronabinol) [67], which was approved for use as an appetite stimulant in patients with AIDS or cancer, but also used under “extra-label” guidelines as an analgesic.

More than half of US states have passed legislation permitting the medicinal use of cannabis in humans under strict guidelines. Additional states have passed laws permitting its recreational drug use.

However, at this time, there are no cannabis-derived products approved by the USFDA for use in animals. Although, the aforementioned products approved by USFDA for human use can be used in animals by veterinarians under the Animal Medicinal Drug Use Clarification Act—AMDUCA of 1994, [68] products that have not been approved by USFDA (which includes the majority of products currently being marketed) are not available for use under AMDUCA.

#### 5.2.2. Authorisation as Food Ingredient or Additive

Due to the considerable expense associated with pursuing USFDA approval for drugs, there is often motivation to, instead, bring such products to the market as food ingredients or food additives. There have been three hemp-derived products approved for use in human food: hemp seed oil, hemp seed protein, and de-hulled hemp seed [69]. However, these products have been approved solely for use in human food and not for use in animal food (including pet foods and treats). At this time, no cannabis-derived products have been approved for use in food for animals. Although an application for the use of hemp seed cake and hemp seed meal as an animal feed ingredient for commercial laying hens has been submitted for review by the American Association of Feed Control Officials (AAFCO) and USFDA, it has not been yet approved [70].

The marketing of cannabis-derived products as supplements raises additional questions. Unlike in the EU, US regulations pertaining to “dietary supplements” for humans do not apply to products intended for use in animals. Products marketed as animal supplements are accordingly regulated as either foods or drugs (not as dietary supplements) depending on their intended use [71]. If their intended use is therapeutic, as indicated by a therapeutic claim or the circumstances of their use, such products are regulated as drugs and must meet USFDA approval to be legally marketed; otherwise, they are regulated as foods. However, under US federal law, foods cannot contain substances that are the active ingredients in approved pharmaceuticals. US federal authorities have concluded that, because THC and CBD are active ingredients in approved drugs, they cannot be incorporated into food [7]. Human dietary supplements also cannot contain substances that are the active ingredients in approved pharmaceuticals, including THC and/or CBD. Accordingly, as of this writing, there are no hemp- or marijuana-derived foods, drugs, or supplements approved for use in animals in the US.

Certain US states, however, have passed legislation that may impact the use of cannabis derived products in veterinary patients. For instance, the US state of Vermont has enacted legislation defining hemp to include products intended for animals. Additionally, US state of Nevada has enacted legislation regarding the manufacturing and marketing of hemp-derived products for animals and in US California state the State Board of Veterinary Medicine has established guidelines for the veterinary discussions of such products [72,73,74]. Most recently, in US state Michigan, legislation was approved allowing veterinarians to consult with animal owners on the use of marijuana or industrial hemp for their animals [75].

### 5.3. North America: Canada

As in the EU and US, certain cannabis-derived substances or their synthetic analogues are available as pharmaceuticals approved for use in humans (see Table 1).

Cannabis-derived veterinary medicinal products that are approved are named veterinary health products (VHP). These veterinary products are listed on the Canadian government website, Health Canada, and are all low risk i.e., no detectable levels of CBD and THC levels < 10 ppm (mg/kg). The list includes several hemp products. VHPs may contain cannabis provided they meet the following criteria, as defined under List C: they are derived from the non-viable seed as a dried or as an extract preparation, concentrations < 10 ppm (mg/kg) of THC, only for use in cats, dogs and horses not intended for human consumption and only for oral and topical route of administration. Any new VHP containing cannabis that meets the List C parameters needs to be notified under the Notification Program.

The Natural Health Products Regulations (NHPR) have been updated to align with the definition of cannabis in the “Cannabis Act” and provide greater clarity about the parts of the cannabis plant that can be included in National Health Products (NHP). Under the updated regulations [76], which came into effect on October 17th, 2018, NHPs can only contain cannabis parts which either do not meet the definition of cannabis in the “Cannabis Act”, or that have been exempted from the “Cannabis Act” through the “Industrial Hemp Regulations” (IHR) [77]. These ingredients are non-viable seeds or roots of the cannabis plant, mature cannabis stalks without leaves, flowers, seeds, or branches, fibre derived from such a stalk and hemp derivatives that are compliant with the IHR. In addition, the above ingredients must contain <10 ppm (mg/kg) THC, or phytocannabinoids that have been isolated or concentrated. The determination of THC concentration must take into account the potential to convert the acid form into the active form (e.g., delta-9-tetrahydrocannabinolic acid (THCA) into delta-9-tetrahydrocannabinol (THC)). Any previously approved NHPs (e.g., hemp seed, hemp seed oil, hemp seed protein) would be unaffected by the transition to the new legislative framework and can continue to be marketed as they are now. Applications for NHPs that contain ingredients compliant with the above requirements would be reviewed under the requirements of the Food and Drugs Act and the NHPR.

Livestock feeds are regulated, as per the Federal Feeds Act and Regulations, by the Canadian Food Inspection Agency (CFIA). All single ingredient feeds manufactured, sold or imported into Canada must be approved and listed in either Schedule IV or Schedule V of the Feeds Regulations. At the present time, hemp products are not approved as livestock feed ingredients in Canada. Each hemp product intended to be used as a livestock single ingredient feed (e.g., hemp meal, hemp oil, seeds) requires separate approval [78].

## 6. Pharmacokinetics/Toxicokinetics

Pharmacokinetics/toxicokinetic parameters are important to consider when evaluating the potential for efficacy/toxicity with varying dose regime (doses, interval, and duration of administration). CBD is a small molecule with a molecular weight of 314.2 g/mol. CBD is highly lipophilic, which raises concerns over potential long-term tissue build-up and toxicity. After a dose of 45 mg of CBD was administered intravenously [79], it was rapidly distributed followed by a terminal plasma half-life (T_1/2β_) of 9 h and a total body clearance (CL) of 0.017 L/h/kg. This study also gave six dogs orally 180 mg of CBD resulting in no analytical detection of CBD in the plasma in three dogs, and an oral bioavailability ranging from 13% to 19% in the other three dogs. The low bioavailability of CBD is due to an extensive first pass or presystemic metabolism in the liver in which CBD and its metabolites are mostly excreted via the kidneys [80,81]. The presystemic metabolism restrains the systemic exposure (i.e., CBD is greatly reduced before it reaches the systemic circulation).

Another dog study [82] administered a cannabis oil extract Bedrocan^®^ (Bedrocan, Veendam, the Netherlands) (20% delta-9-tetrahydrocannabinol (THC) and 0.5% cannabidiol (CBD)) in fasting and fed dogs at 1.5 and 0.037 mg/kg THC and CBD. Blood samples (1 mL) were withdrawn at 5, 15, 30, 45 min and 1, 1.5, 2, 4, 6, 8, 10, 24, 36, 48, 72 and 96 h after administration of Bedrocan^®^. No analytical detectable concentrations of CDB were found at any collection time. THC was quantifiable from 0.5 to 10 h, although there was large inter-subject variability. Fed dogs showed a longer absorption phase (Tmax 5 versus 1.25 h) and lower maximal blood concentration (7.1 versus 24 ng/mL) compared with the fasted group. THC is a lipophilic compound and should have increased bioavailability in the fed condition (bioavailability: 48.22%; fasted no reported) [82].

In humans, a study on the food effect on the pharmacokinetics of cannabidiol oral capsules administered with and without food in adults with refractory epilepsy, showed that the fat content of a meal can lead to significant increases in the peak serum concentration (Cmax) (14 times higher in the fed state compared to fasting) and area under the plasma concentration-time curve (AUC_0-u_) (4 times higher in the fed state compared to fasting), Tmax was found to be highly variable within states with the fasting state Tmax ranging from 2 to 5 h and 1 to 6 h for the fed state and therefore can account for variability in bioavailability. Blood samples were collected from an indwelling catheter or venipuncture. During both fed and fasting states, blood samples were collected at predose and at 0.5, 1, 2, 2.5, 3.5, 4, 5, 6, 24, 48, and 72 h. CB doses were 200 mg for one subject and 300 mg for all others during the fed and fasting sessions. Steady state concentrations post 300 mg once daily dose ranged from 4.73 to 40 ng/mL with an average of 21.33 ng/mL [83].

In another pharmacokinetics study, each dog was injected intravenously (cephalic vein) with CBD (90 mg in 2 mL of 70% ethanol). After dosing, the urine volumes were continuously collected for 30 h at intervals of 0, 1, 2, 3, 4, 6, 8, 10, 12, 14, 22, 26, and 30 h. The apparent terminal plasma half-life (T_1/2β_) of CBD from the urine data was significantly shorter than the elimination half-life (T_1/2_) of CBD calculated from the plasma data (7–9 h). The T_1/2_ of all the metabolites was similar to that of CBD indicating that the excretion of these urinary metabolites was formation-rate limited [84].

In dogs, the onset of clinical signs in cases of marijuana toxicosis typically occurs within 30–90 min of exposure and can last up to 96 h [85]. After oral ingestion, THC is almost completely absorbed. THC goes through a substantial first-pass or presystemic metabolism. It is metabolized by liver microsomal hydroxylation and non-microsomal oxidation. THC is highly lipophilic and readily distributes to the brain and other fatty tissues following absorption. Its high lipid solubility contributes to its large volume of distribution (Vd) and long elimination half-life (T_1/2_). One pharmacokinetic study investigated the use of a phytocannabinoid-based medicine—Sativex^®^ (GW Pharma Ltd., Sovereign House, Vision Park, Histon, Cambridge, UK) in dogs which is currently marketed for the treatment of spasticity and pain of multiple sclerosis in humans. It was shown that single or multiple doses administered sublingually to dogs resulted in maximum plasma concentrations of phytocannabinoids at 1–2 h and suggested progressive accumulation after the multiple dose treatment [86]. Another study carried out in dogs found non-proportional increases in plasma cannabinoid concentrations with increasing oral doses, this as well as potential differences in Cannabis herbal extract product composition and dose regimen consistency, which may all lead to adverse effects [87]. One study showed adverse events associated with CBD administration including elevation in liver enzymes (*n* = 14) and vomiting (*n* = 2) [88].

Finally, it was stated that most pharmaceuticals follow a familiar pharmacokinetics pattern by demonstrating a linear dose–response curve and thus displaying a direct linear relationship between the increasing dose and increased efficacy until a maximum level of efficacy is reached. The dosing of cannabis above this maximal effect may lead to an increase in adverse effects with little to no increase in therapeutic value [18].

### 6.1. Mechanism of Action

A major advance in our understanding of how cannabis works has been the discovery of specific receptor proteins in the brain that recognize cannabinoids including THC.

Among several that have been identified, the two primary types of cannabinoid receptors are CB1 and CB2, both coupled to G-proteins [19]. CB1 and CB2 receptors have been identified in rats, guinea pigs, dogs, monkeys, pigs, and humans [89]. CB1 receptors are widely distributed in the brain (central nervous system—CNS) and correlate with cannabinoid effects on cognition, appetite, emotions, memory, perception and control of movement. An interspecies variation in the anatomical location of the CB1 receptors is seen in dogs. Dogs in particular have a higher density of CB1 receptors in their cerebellum compared to any other species studied [90]. The existence of endogenous cannabinoid receptor agonists has also been demonstrated. These discoveries have led to the development of selective cannabinoid CB1 and CB2 receptor ligands.

CB1 receptors are located within lipid membranes of presynaptic neurons. They inhibit cAMP and stimulate mitogen-activated protein kinases to modulate the control of ion channels, particularly voltage-activated calcium ion channels and potassium channels. The result is the inhibition of the neurotransmitter release, both excitatory and inhibitory. CB1 receptors also activate phospholipase C and PI-3-kinase. The endogenous ligand for cannabinoid receptors, known as endocannabinoids, are derived from arachidonic acids and are closely related to prostaglandins.

CB2 receptors are less frequently found in the CNS but are highly concentrated in the peripheral nervous system and immune system where they play a part in inflammation and pain regulation [91]. CB2 receptors regulate ceramide biosynthesis. The endogenous cannabinoids bind to these receptors as part of the modulation of signalling pathways and in association with several pathophysiological conditions (e.g., neurological disorders).

### 6.2. Pathogenesis

CBD is believed to act on unique receptors in the brain that are selective for cannabinoids and responsible for the CNS effects. Cannabinoids can enhance the formation of norepinephrine, dopamine, and serotonin. Toxic effects are an extension of the pharmacological and clinical effects. The oral LD_50_ values of pure THC in rats and mice are 666 and 482 mg/kg body weight, respectively [56]. The minimum lethal oral dose in the dog for THC is greater than 3 g of plant material per kg body weight [92].

## 7. Use in Human and Veterinary Medicine

### 7.1. Use in Human Medicine

The effects of cannabis on pain mechanisms are probably responsible for the medical interest in this substance. In human medicine, cannabinoids such as CBD and THC are being used for the treatment of epilepsy and the management of pain and inflammation associated with osteoarthritis and spasms associated with multiple sclerosis (MS) [32]. A non-exhaustive list of medicinal products approved for human use under strict conditions can be found in Table 1.

### 7.2. Use in Veterinary Medicine

At the time of writing, there are no authorized cannabis or cannabis-derived veterinary medicinal products on the market in the EU, US, or Canada. On the EU market, one product is authorised in Germany (Cardio ReVet RV4) and registered as a homeopathic veterinary medicine [93]. In addition, one CBD product (Anibidiol) registered as a feed supplement is marketed in several EU Member States such as France and the Netherlands [94]. Anibidiol contains CBD and Vitamins B3 and B6. The leaflet mentions “*Veterinarians have good experience in using this product for behavioural problems, pain, infection, epilepsy and the consequences of tumours in dogs and cats*”. The product is available without the need for a veterinary prescription.

Veterinarians could potentially use cannabis products authorised for human use “off-label” in animals. The relevant legal text is detailed in Articles 10 and 11 of the EU directive 2001/82/EC, (known as “the cascade”) and the AMDUCA in the US [68,95]. It is the responsibility of the veterinarian to understand their legal obligations.

Available scientific evidence pertaining to their use in animals is currently limited and focused on companion animals and horses. Some findings from a few well-controlled clinical studies have been published, other information is gleaned from anecdotal support, historical records, and case reports or has been extrapolated from studies related to use in humans, including the study of animal models for that purpose.

Areas of interest include use for osteoarthritis pain [88,96,97,98], other types of pain (oncologic, neuropathic) [99], immune-mediated and inflammatory allergic disorders [100], cardio-vascular and respiratory conditions [101], and epilepsy [102,103]. According to the scientific literature review, cannabinoids are mainly used in the treatment of pain, especially osteoarthritis pain.

It is difficult to estimate the real efficacy of using cannabinoids, as shown by the outcome of the underneath Table 2 showing a selection of studies investigating the effect of CBD oil on osteoarthritis in dogs.

The use of cannabinoids in the treatment of epilepsy in dogs was discussed in another clinical study. In this study, cannabinoids were given as an adjunct therapy to antiepileptic therapy for 12 dogs. Two dogs in the CBD group developed ataxia and had to be withdrawn. In the end, nine dogs in the CBD group were compared with seven in the placebo group. Reduced seizure frequencies were observed in the CBD group, however, the proportion of dogs considered responders to treatment (≥50% decrease in seizure activity) was similar between groups. The dogs showed no detectable analytical plasma CBD concentrations at any assessment point [103].

There is also interest in using CBD and other cannabis-derived products for horses, e.g., against arthritis and negative stereotypical behaviours in horses [104]. The Fédération Equestre Internationale (FEI), the international government body for equine sports, has declared all cannabinoids as banned substances on its Equine Prohibited Substances List [105]. Products that cannot be given to competition horses include natural and synthetic cannabinoids and other cannabimimetics.

## 8. Concerns Regarding Illegal Product Claims and Unknown Composition

CBD oil products may be marketed that do not conform to regulations, both animal and human. In the US, the FDA has also had to cite multiple companies illegally selling CBD products because the companies claimed they could prevent, diagnose, treat, or cure disease with some of these companies marketing products targeted toward animals [106].

The marketing of illegitimate products occurs, particularly when the regulatory framework is not well known and during periods of the rapid expansion of any particular sector. In the EU, all labelling information for CBD feed additives is controlled by EFSA for animal feed and human food, under the Regulation (EU) No 1169/2011 [107], (EC) No 178/2002 [55], and Regulation (EC) No 1924/2006 [63]. The principles behind the legislation are the 100% accuracy of labelling and claims based on scientific evidence. Veterinarians may be the most likely professional group to observe abnormal reactions, for example, psychoactive responses in animals that may have received a defective product (see Section 9). Veterinarians may also be approached to stock CBD products in their clinics.

In all regions, the inaccurate labelling of the identity and strength of active ingredients is of particular concern, making administration according to a dosage regimen very difficult to impossible [108]. In Europe, only the composition of medical cannabis is known and controlled [30]. In the US, the composition of FDA-approved therapeutic products for humans is known and controlled, but less consistency in quality assurance exists around cannabis sold for medicinal purposes by dispensaries regulated at the state and local levels [109]. Only a few US states have extensive product tracing programs where specific testing methods to confirm the label accuracy of the intended chemical components as well as unintended contaminants are established. Much variation exists in which intended and unintended chemical constituents are analysed, as well as in methods of analysis [110]. Therefore, the label accuracy of cannabinoid constituents and potential contaminants for non-FDA-approved cannabis products may vary considerably within and among US states. Few studies have been done on the safety and tolerability of these products in animals [111,112]. Several studies have shown quality and safety issues related to the use of products, often illegal, labelled for animal use [113,114], some of which have been associated with toxicoses.

## 9. Cannabis Toxicosis in Veterinary Medicine

Veterinary cases of cannabis toxicosis in dogs stem most commonly from exposure to edibles. In these cases, there may be additional toxic ingredients involved—such as chocolate, raisins, or xylitol—which result in a poorer prognosis. Cats may also directly consume the plant material.

The likelihood of pets becoming exposed is increasing as cannabis and cannabis products become more widely available and recreational drug use more commonplace. A US study in 2012 reported increased rates of toxicosis seen in dogs living in Colorado, a state in which cannabis had been recently legalized for human medicinal use; in fact, a four-fold increase in toxicoses was reported between 2005 and 2010 [115]. In 2019, the US ASPCA’s Animal Poison Control Center noted a large jump in calls about marijuana ingestion by animals; a 765% increase in the first few months over the same period the previous year. An increase was seen even in US states where cannabis has not yet been legalized [116]. This pattern may be a result of the general shift towards cultural acceptance of marijuana use, and the growing availability and use of marijuana edibles, the leading cause of intoxication in dogs.

There is currently little research performed on thresholds for toxicosis. Although the available data suggest that CBD may be well tolerated by animals and produces few side effects, the lack of industry-wide quality control can result in an animal’s exposure to hemp or CBD products contaminated with THC or toxins, such as heavy metals or pesticides, that may cause harm. In the US, animal poison control organizations indicate that up to 50% of pets exposed to products labelled as CBD or hemp may develop clinical signs severe enough to require veterinary intervention, indicating that such products may not be pure CBD [117]. Several deaths were reported to have been related to cannabis toxicoses, and these appear to be the result of associated complications, such as aspiration.

In dogs, excessive THC intake can easily result in clinical signs of toxicosis. Synthetic cannabinoids can have a higher potency than THC. Clinical signs of anxiety, hallucinations, seizures, psychosis, and tachycardia are reported in patients but most recover within several hours [118]. Smaller dogs are particularly susceptible due to the smaller amount required to produce clinical signs. Cats are not immune to toxic side effects but are much more selective in their food intake and do not appear to consume cannabis edibles as often as dogs. Cats, as a species, generally avoid eating garbage and scavenging cigarette butts, or table, or counter surfing in comparison to dogs. They also seem to be less attracted to products with a high concentration of sugars, so we do not see them take in baked good “pot” products like dogs typically do. One experimental study of cannabis exposure in cats showed that, cannabinoids caused bradycardia, hypotension and respiratory depression, which depending on the chemical composition of the product and the exposure amount, may be expected in the case of intoxication [101].

### 9.1. Clinical Signs

A wide range of clinical signs have been associated with cannabis toxicosis. A classic presentation is a depressed or ataxic dog that is dribbling urine. Further clinical signs are listed in Table 3. Most of them are neurological signs.

### 9.2. Diagnosis

Cannabis toxicosis can look similar to intoxication with numerous other sedatives, with the most common and serious of those being anti-freeze poisoning (i.e., ethylene glycol) or ivermectin toxicosis. In most cases, the diagnosis can be made based on the history of exposure, anamnesis and clinical signs. Cannabinoids are difficult to detect in body fluids, particularly because of their high lipid solubility and low concentrations found in urine and plasma. Urine testing can be performed but can give false negatives. The confirmation of positive screening tests requires the use of chromatographic analytical techniques (GLC, HPLC, TLC, GC–MS) that are capable of separating and detecting the major metabolites. GC–MS is the most reliable confirmatory method, especially when used with electron impact (EI) and chemical ionization (CI) detector modes. Common adulterants that mask a positive test for marijuana metabolites include detergents, salt, use of diuretics, and vinegar [92,119].

### 9.3. Treatment and Prognosis

Because no antidote has been described to date, the treatment of cannabis toxicosis consists of supportive care [116,120,121,122]. Because of the wide margin of safety of most known cannabinoids, toxicosis is rarely fatal. Steps that should be taken include:

- If less than 30 min have passed since consumption, the animal should be decontaminated by inducing emesis and administering activated charcoal and cathartic. Repeated dosing with activated charcoal and cathartic may reduce the elimination half-life of THC by interrupting enterohepatic recirculation.
- If clinical signs have started, inducing emesis might be difficult (due to the psychoactive properties of THC) and could be dangerous if the patient is heavily sedated, as vomit could be inhaled and lead to aspiration pneumonia.
- Fluid support and keeping the patient warm may also be needed due to hypothermia. The patient should be rotated frequently to prevent dependent oedema or decubital ulceration.
- Diazepam can be given for sedation or to control seizures.
- Administer oxygen to assist respiration or relieve respiratory depression, if needed.
- Treat central nervous system depression, if needed.
- IV lipid emulsion therapy may be helpful in the treatment of severe cases [122,123].
- If the patient has lost consciousness, intense observation and support are needed. The chance of fatality is statistically small but possible.
- Recovery may take 24 to 72 h, or longer (up to 5 days), depending on the ingested dose.

## 10. Conclusions and Recommendations

Further research is recommended to improve our understanding of the safety and effectiveness of the use of cannabis-derived products in veterinary medicine. Current research is limited, mostly done on small samples and at times with conflicting outcomes. This suggests that cannabinoid products may potentially be beneficial in certain cases to reduce pain, particularly osteoarthritis pain, and as an adjunctive treatment of canine epilepsy. Currently, no cannabis-derived veterinary medicinal products are authorised in the EU or North America. The off-label use of human medicinal products might be allowed to be used in animals in certain EU countries or in the US, only when using EU or USFDA-approved products, respectively. It is the responsibility of the veterinarian to understand their legal obligations.

For the future we recommend the following initiatives:

- Well-controlled clinical trials (double-blinded, placebo controlled) and pursuit of EU/North American approval or approval at the national level by manufacturers of cannabis-derived products should be conducted, so that high-quality products of known safety and efficacy can be made available for veterinarians and their patients.
- Clinical trial studies should be encouraged to investigate the potential therapeutic value and safety of hemp-derived products for companion animals.
- Harmonize the analytical procedure of the determination of the THC level in serum and oral fluids and set up harmonised tolerable limits of cannabinoids in different products.
- Use of hemp-derived products for animals should require a veterinary prescription.
- Prohibition on producing pet food with cannabis-derived products without known safety and efficacy and without the knowledge of the intended purpose of the included cannabis-derived products as specified by the pet food manufacturers.
- The prohibition on producing feed supplements or beddings for food producing animals with CBD/Cannabis without known safety and efficacy and without knowledge of the intended purpose of the included cannabis-derived products as specified by manufacturers, and data on any residue in the food derived from these animals.
- We encourage veterinarians to act cautiously, as there may be risks associated with having such products in their possession if the product(s) were subsequently shown to contain illegal levels of THC. Any suspected breaches should be reported to Competent Authorities in the EU Member State where the event occurred.

Greater international cooperation is needed to help define standards, promote safety, education, research, and policy.

## Figures and Tables

**Table 1:** Non-exhaustive examples of authorised cannabis-based medicines in Europe and North America at a glance.

Brand Name	Pharmaceutical Form	Active Substance	Clinical Indication	Approved In
Sativex (Nabiximols)	Oromucosal Spray	δ-9 THC and CBD from *Cannabis sativa* L.	Multiple sclerosis	Canada, EU
Marinol (Dronabinol)	Gelatin capsule	Synthetic δ-9-THC	Nausea with cancer treatment, anorexia with AIDS, multiple sclerosis	EU, US
Cesamet (Nabilone)	Capsule	Synthetic cannabinoid similar to THC	Cancer treatment	Canada, EU, US
Epidyolex/Epidiolex	Liquid to be taken orally	CBD	Against epilepsy	EU, US

**Table 2:** Non-exhaustive listing of CBD studies on dogs and results.

Species (Number of Animals)	Dose/Time of Exposure	Objective	Result/Conclusion	Reference
Dogs (37)	CBD oil product at a dose of 0.25 mg/kg delivered in food, once a day for 3 days and then morning and night (approximately every 12 h).	To assess the impact of a full-spectrum product containing hemp extract and hemp seed oil on dogs with chronic maladaptive pain.	Thirty of the 32 dogs showed a significant improvement in pain reduction. The addition of a hemp-derived CBD oil appears to positively affect dogs with chronic maladaptive pain (decreasing their pain and improving their mobility and quality of life).	Kogan et al., (2020) [96]
Dogs (16)	CBD oil (2 and 8 mg/kg) or placebo oil every 12 h.	To determine basic oral pharmacokinetics and assess safety and analgesic efficacy of CBD-based oil in dogs with osteoarthritis.	Pharmacokinetics revealed an elimination half-life (T_1/2_) of 4.2 h. at doses of 2 and 8 mg/kg. The peak serum concentrations of CBD oil 102.3 ng/mL and 590.8 ng/mL occurred at 1.5 and 2 h, respectively, for 2 and 8 mg/kg doses. CBD produced a significant decrease in pain and an increase in activity in the group treated with CBD	Gamble et al., (2018) [97]
Dogs (23)	CBD treatment for 6 week (2.5 mg/kg per dog every 12 h).	To provide preliminary data describing the safety and effect of CBD for clinical signs relief of canine osteoarthritis associated.	Baseline data were acquired for 4 weeks, followed by the random allocation to the placebo group or CBD treatment group for 6 weeks, followed by 6 weeks with the opposite treatment. No differences were found between groups at any time point for any of the recorded outcome measures. Adverse effects associated with CBD administration included enzymes liver elevations and vomiting signs.	Mejia et al., (2021) [88]
Dogs (20)	Four groups: placebo, 20 mg/day (0.5 mg/kg) naked CBD, 50 mg/day (1.2 mg/kg) naked CBD, or 20 mg/day liposomal CBD.	To support the safety and therapeutic potential of hemp-derived CBC for relieving arthritic pain.	CBD significantly decreased pain and increased mobility. Liposomal CBD (20 mg/day) was as effective as the highest dose of non-liposomal CBD (50 mg/day) in improving clinical outcome. It was demonstrated that the widely available supplement CBD exerts robust and quantifiable anti-inflammatory properties in experimental systems.	Verrico et al. 2020 [98]

**Table 3:** The most common signs of excess cannabis exposure in dogs and cats.

Neurological	Sleepiness
	Ataxia
	Depression
	Wobbling, pacing and agitation
	Vocalization
Eyes	Dilated pupils
	Bloodshot eyes
Gastro-intestinal	Vomiting
	Salivation
Others	Sound or light sensitivity
	Inappropriate urination
	Fast or slow heart rates
	Low body temperature

## Data Availability

No new data were created or analyzed in this study. Data sharing is not applicable to this article.
